# Utility of Surface Pollen Assemblages to Delimit Eastern Eurasian Steppe Types

**DOI:** 10.1371/journal.pone.0119412

**Published:** 2015-03-12

**Authors:** Feng Qin, Yu-Fei Wang, David K. Ferguson, Wen-Li Chen, Ya-Meng Li, Zhe Cai, Qing Wang, Hong-Zhen Ma, Cheng-Sen Li

**Affiliations:** 1 State Key Laboratory of Systematic and Evolutionary Botany, Institute of Botany, Chinese Academy of Sciences, Nanxincun 20, Xiangshan, Beijing, 100093, China; 2 Institute of Palaeontology, University of Vienna, Althanstrasse 14, Vienna, A-1090, Austria; 3 Institute of Geology and Paleontology, Linyi University, Linyi, 276005, China; 4 Beijing Radiation Center, Beijing Academy of Science and Technology, Beijing, 100875, China; 5 College of Landscape Architecture, Beijing Forestry University, Beijing, 100083, China; 6 Key Laboratory of Land Surface Pattern and Simulation, Institute of Geographic Sciences and Nature Resources Research, Chinese Academy of Sciences, Beijing, 100101, China; USDA-ARS, UNITED STATES

## Abstract

Modern pollen records have been used to successfully distinguish between specific prairie types in North America. Whether the pollen records can be used to detect the occurrence of Eurasian steppe, or even to further delimit various steppe types was until now unclear. Here we characterized modern pollen assemblages of meadow steppe, typical steppe and desert steppe from eastern Eurasia along an ecological humidity gradient. The multivariate ordination of the pollen data indicated that Eurasian steppe types could be clearly differentiated. The different steppe types could be distinguished primarily by xerophilous elements in the pollen assemblages. Redundancy analysis indicated that the relative abundances of *Ephedra*, *Tamarix*, *Nitraria* and Zygophyllaceae were positively correlated with aridity. The relative abundances of *Ephedra* increased from meadow steppe to typical steppe and desert steppe. *Tamarix* and Zygophyllaceae were found in both typical steppe and desert steppe, but not in meadow steppe. *Nitraria* was only found in desert steppe. The relative abundances of xerophilous elements were greater in desert steppe than in typical steppe. These findings indicate that Eurasian steppe types can be differentiated based on recent pollen rain.

## Introduction

Pollen data may be used to characterize current [e.g. 1–5] and ancient [e.g. 6–10] plant communities. Modern pollen assemblages have been used to distinguish grassland types [e.g. 11–16]. For example, Hoyt [12] determined the main floral elements represented as pollen were useful for distinguishing prairie types in the Great Plains, North America. They determined that prairie could be clearly differentiated from forest, and four types of prairie (i.e. tall-grass, mixed-grass, short-grass and desert grasslands) could also be distinguished from each other using pollen data.

**Fig 1:**
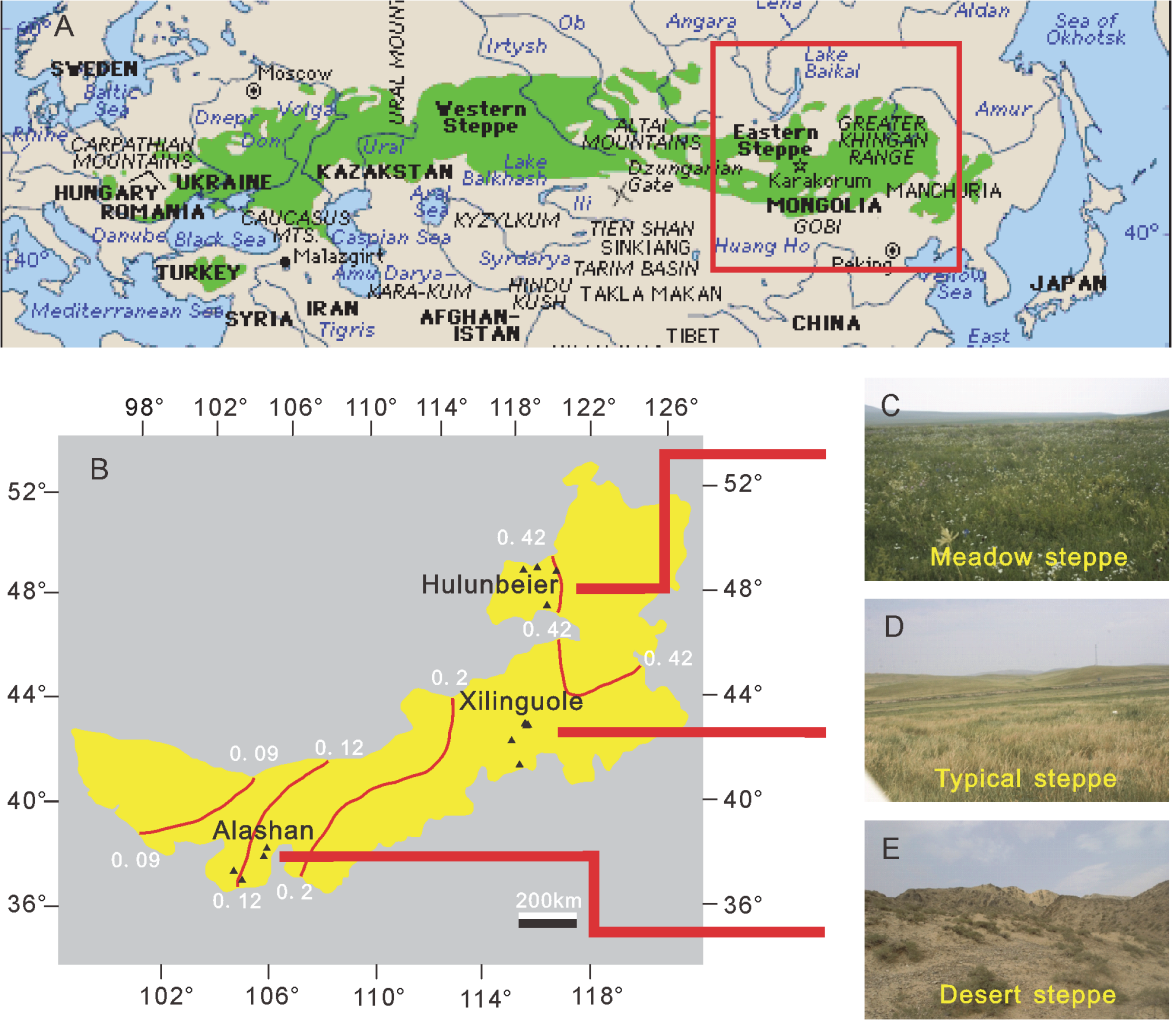
Maps showing the sampling localities of 3 steppe types in Inner Mongolia, eastern Eurasia. A. Map showing the extent of the Eurasian steppe from http://davidderrick.files.wordpress.com. B. Map showing the extent of Inner Mongolia, and the surface soil sampling sites (▲); red lines are the isolines of mean annual humidity (redrawn from [30]). Scale bar represents 200 km. C, D and E represent images of meadow steppe, typical steppe and desert steppe respectively.

However, similar studies are lacking for the Eurasian steppe which extends across mid-latitudes of Eurasia (Fig. 1). Three types of steppe can be recognized along the humidity gradient in Inner Mongolia, northern China, i.e. meadow steppe, typical steppe and desert steppe [17]. Many papers on the modern pollen assemblages in steppes of Inner Mongolia, northern China are available [13,15,16,18–22]. However, most of them only dealt with the pollen assemblages of one or two steppe types [13,15,18–22], and none of them distinguished all three types of steppe along the humidity gradient. It is unclear whether the pollen assemblages from the surface soil of Eurasia can be used to detect the presence of steppe, or to distinguish the three types of steppe.

In traditional interpretation of pollen data, Chenopodiaceae/*Artemisia* (C/A) ratio is frequently used as a semi-quantitative aridity proxy in arid and semiarid region [e.g. 23–27]. El-moslimany [24] investigated the surface pollen assemblages in the Middle East along an aridity gradient. He noticed that the C/A ratio increases with the intensification of aridity. After that discovery, the C/A ratio became widely used as an aridity proxy by palynologists [e.g. 23,25–27]. In eastern Asia, several studies [13,16,18,19] suggested that the C/A ratio of surface pollen is a good indicator for aridity changes in the Inner Mongolian steppe. However, this viewpoint was challenged by other regional studies [e.g. 15,20,28]. In a recent review on the C/A ratio, Zhao et al. [29] proposed that the C/A ratio can only be used in regions with precipitation < 450–500 mm, and discussed the influences of soil salinity, vegetation composition, human activity and sample provenance on the C/A ratio. More research is needed to explore the relationship between C/A ratio and aridity in steppe.

Here we investigated the surface pollen assemblages of meadow steppe, typical steppe and desert steppe along the ecological humidity gradient in Inner Mongolia (Fig. 1). We hypothesized that variation in steppe communities would be explained by environmental variables (e.g. mean annual temperature, mean annual precipitation), and attempted to differentiate steppe types. We also sought to characterize the relationship between C/A ratio and aridity, and provide additional empirical evidence on the importance of this as an arid indicator in Eurasian steppe.

## Materials and Methods

Surface pollen samples were collected from natural plots (far from human settlements) in 3 different types of steppe along the humidity gradient in Inner Mongolia. The mean annual precipitations (MAP) of the three steppe types are 350–500 mm, 200–400 mm, and 150–280 mm respectively, while the annual humidities (ratio of annual precipitation to annual potential evapotranspiration) of these areas are >0.4, 0.2–0.4, and 0.12–0.2 [30]. We sampled 4 meadow steppe sites at Hulunbeier (48°00′20″–49°27′26″N, 117°34′40″–119°29′34″E, 620–893m), 6 typical steppe sites at Xilinguole (42°02′40″–43°39′44″N, 116°02′39″–117°08′52″E, 1243–1435m) and 4 desert steppe sites at Alasha (37°43′26″–38°58′48″N, 104°35′23.58″–105°52′23.38″E, 1372–1972m) (Fig. 1). We collected 5 surface soil subsamples per site which were then mixed into a single composite sample per site. Since it was unclear whether steppe types could be differentiated from forest based on pollen data, we also sampled a regional forest which enabled us to compare the pollen assemblages of steppe and forest. A pollen survey of a typical warm temperate forest on Dongling Mountain in Beijing, northern China (MAP 612 mm [31]) was carried out. A composite sample of surface soil and a record of airborne pollen during 2008–2011 were collected for pollen analysis at this site (39°57′34″–39°58′4″ N, 115°25′42″–115°25′53″ E, 1143–1207 m a.s.l.).

**Fig 2:**
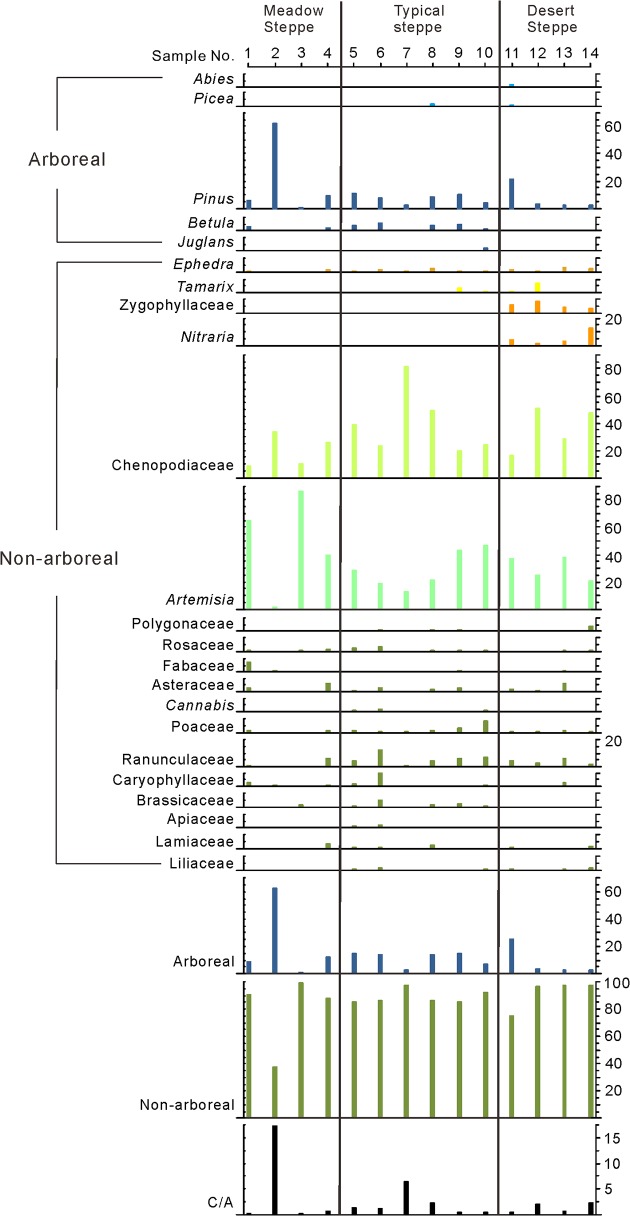
Surface pollen spectra of meadow steppe, typical steppe and desert steppe in Inner Mongolia, China.

The samples were treated by the method of Heavy Liquid Separation (Density = 2.0g/ml) [32,33]. Pollen and spores were observed and counted using a Leica DM 2500 light microscope (LM), and identified by referring to the palynological literature [34–38]. More than 300 grains (304–616) of pollen and spores per sample were counted. Tilia 1.7.16 [39] was used to construct the pollen diagram (Fig. 2).

The assignment to the angiosperm families in this article largely follows APG III [40,41]. One exception is the Chenopodiaceae, which was retained even though APG III have assigned the plants from former Chenopodiaceae to the family Amaranthaceae, since the Chenopodiaceae was frequently used as an aridity indicator in palynological studies [e.g. 24,29].

The taxonomic list of the Inner Mongolian steppe is based on Wu [42] and ISTIMN [17], and that of the warm temperate deciduous broad-leaved forest of Dongling Mountain refers to “The list of plant taxa at the Xiaolongmen Area, Beijing, China” which was provided by the Beijing Forestry University as the fieldwork identification handbook. The plant taxa lists were converted to match the taxonomic distinctions made based on pollen morphology (S1 Table and S2 Table).

**Fig 3:**
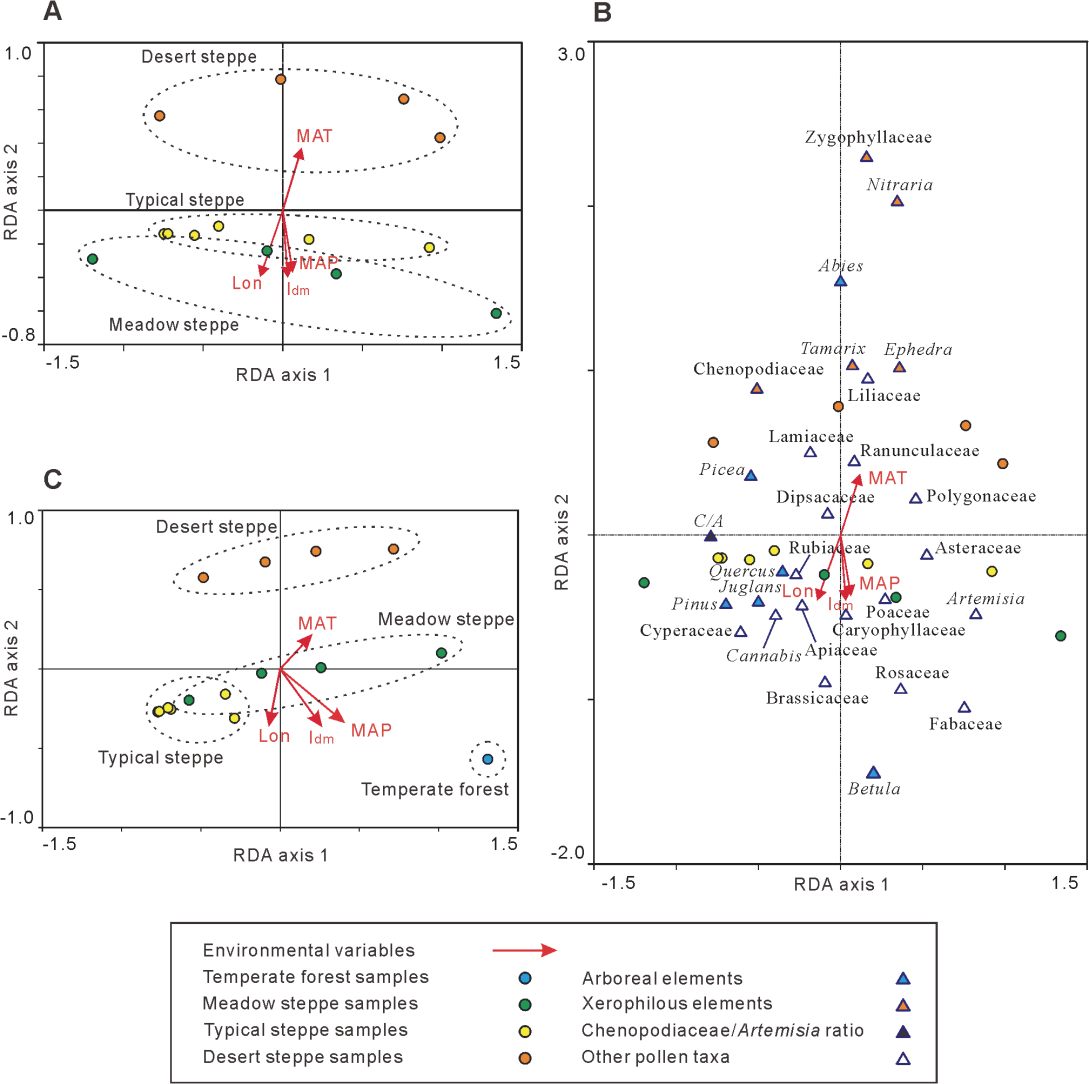
Redundancy analysis (RDA) plots for sampling sites and pollen taxa on axes 1 and 2. A. Ordination biplot of sampling sites (circles) and 4 environmental variables (arrows) for the ordination including only steppe data. B. Ordination triplot of pollen taxa (triangles), sampling sites (circles) and 4 environmental variables (arrows) for the ordination including only steppe data, within which only those pollen taxa with significant relative abundance (>1% in at least one sample) are shown. C. Ordination biplot of sampling sites (circles) and 4 environmental variables (arrows) for the ordination containing both steppe and forest data. MAT, mean annual temperature; MAP, mean annual precipitation; I_dm_, de Martonne aridity index; Lon, longitude.

**Fig 4:**
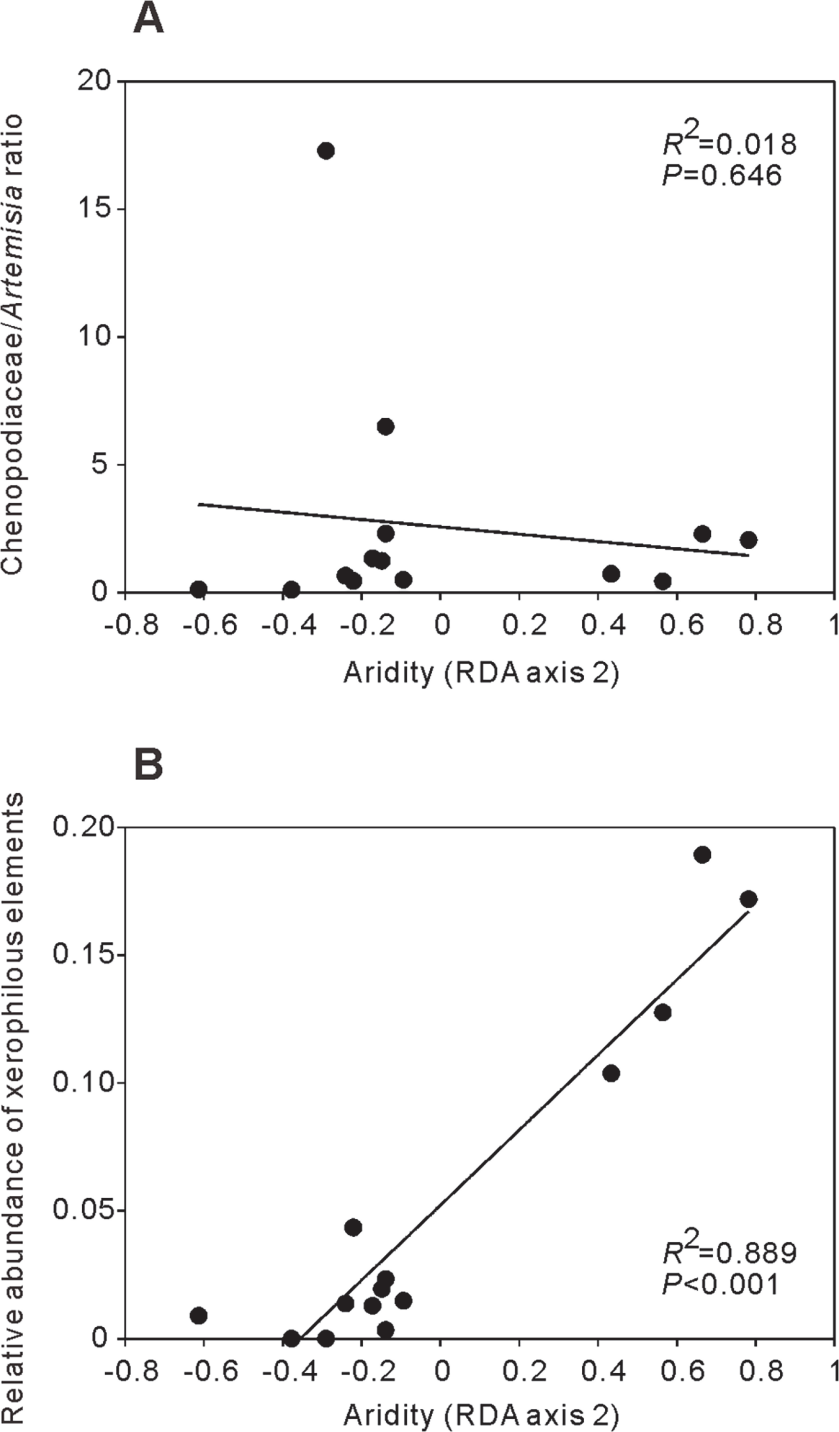
Scatter plots of C/A ratio (A) and the relative abundance of xerophilous elements (B) versus aridity gradient. Results of linear regression are shown (*R* ^*2*^, *P* and fitted lines).

Ordination techniques were used to analyze relationships between pollen data and environmental variables, and similarities among pollen assemblages of different sites. Two ordination procedures were performed, the first of which analyzed the pollen data of steppe, and an additional one was carried out to evaluate both steppe and forest data. A preliminary detrended correspondence analysis (DCA) on pollen data of steppe samples yielded a gradient length of 1.44 standard deviation units (SD) of species turnover on the first axis, while that on both steppe and forest data yielded a gradient length of 1.71 SD. Consequently, linear-based methods such as redundancy analysis (RDA) were suitable for studying the pollen data in both cases [43]. DCA and RDA were processed using CANOCO 4.5, and ordination plots (Fig. 3) were constructed by CANODRAW 4.1 [44]. When processing RDA, pollen percentage data were square-root transformed and centered by taxa, symmetric correlation was chosen, and taxa scores divided by the standard deviation. Mean annual temperature (MAT), mean annual precipitation (MAP), de Martonne aridity index (I_dm_) and longitude were selected as environmental variables. The de Martonne aridity index (I_dm_) [45] was used to estimate aridity changes, which was calculated by the equation: I_dm_ = MAP / (MAT + 10). Low I_dm_ values indicate arid climates. MAP and MAT data of the sampling sites referred to the Earth Systems Modeling Results (http://www.paleo.bris.ac.uk/ummodel/scripts/html_bridge/clamp_UEA.html). In addition, since RDA axes were linear combinations of the selected environmental variables [46], they could be used to evaluate the synthesized impact of environmental variables on surface pollen assemblage. Here, the RDA axis 2 was adopted as an aridity gradient (detailed reasons refer to Results section). Univariate linear regression analyses were performed to examine the relationship between aridity and C/A ratio, xerophilous elements (Fig. 4).

### Ethics Statement

All necessary permits were obtained for the described field studies and were granted by the local government of Inner Mongolia. The field work did not involve endangered or protected species.

## Results

### 1. Pollen assemblages of steppes in Inner Mongolia, northern China

The surface pollen assemblages of the Inner Mongolian steppe yielded 34 palynomorphs, consisting of 27 non-arboreal and 7 arboreal elements (S1 Fig., S3 Table). The relative abundance of non-arboreal pollen was 87.7%, among which *Artemisia* (37.2%) and Chenopodiaceae (31.4%) were the dominant elements. Ranunculaceae (4%), Asteraceae (1.9%), and Poaceae (1.5%) were commonly found. Xerophilous elements such as *Ephedra* (1.2%), *Tamarix* (1%), Zygophyllaceae (1.9%) and *Nitraria* (1.5%) were also detected. The arboreal pollen mainly consisted of *Pinus* (10.1%) and *Betula* (1.6%) (Fig. 2, Table 1).

**Table 1:** The list of the palynomorphs and their relative abundances of 3 steppe types (meadow steppe, typical steppe, and desert steppe) in Inner Mongolia.

**Taxa**	**Relative abundance (%)**				**Taxa**	**Relative abundance (%)**			
	**Inner Mongolia steppe (in general)**	**Meadow steppe**	**Typical steppe**	**Desert steppe**		**Inner Mongolia steppe (in general)**	**Meadow steppe**	**Typical steppe**	**Desert steppe**
**Arboreal**					Fabaceae	0.77	1.73	0.36	0.30
***Gymnosperms***					Rosaceae	0.74	0.66	1.24	0.24
*Pinus*	10.13	16.91	7.15	6.80	*Cannabis*	0.19	0	0.52	0
*Abies*	0.19	0	0	0.59	Polygonaceae	0.49	0.06	0.62	0.77
*Picea*	0.21	0.06	0.26	0.30	Celastraceae	0.02	0	0	0.06
***Angiosperms***					Scrophulariaceae	0.04	0	0.10	0
*Betula*	1.62	1.19	3.37	0.06	Brassicaceae	0.77	0.66	1.55	0
*Castanea*	0.02	0	0	0.06	Apiaceae	0.28	0.06	0.67	0.06
*Quercus*	0.02	0	0.05	0	Lamiaceae	0.66	0.71	0.62	0.65
*Juglans*	0.15	0.06	0.36	0	*Scabiosa*	0.09	0.06	0.16	0.06
**Non- arboreal**					*Viola*	0.06	0	0	0.18
***Pteridophytes***					Rubiaceae	0.11	0	0.31	0
Polypodiaceae	0.06	0.06	0.10	0	Boraginaceae	0.06	0	0.05	0.12
***Gymnosperms***					Zygophyllaceae	1.87	0	0.05	5.80
*Ephedra*	1.15	0.48	1.14	1.83	*Nitraria*	1.51	0	0	4.73
***Angiosperms***					*Tamarix*	0.98	0	0.78	2.19
Chenopodiaceae	31.42	18.34	38.96	35.80	Poaceae	1.49	0.71	2.85	0.71
Asteraceae	1.91	1.91	1.45	2.42	Cyperaceae	0.09	0.06	0.21	0
*Artemisia*	37.16	53.42	28.76	30.59	Liliaceae	0.40	0	0.52	0.65
Ranunculaceae	3.98	1.73	5.85	4.08	*Typha*	0.02	0.06	0	0
Caryophyllaceae	1.34	1.07	1.92	0.95					

#### Meadow steppe

21 types of palynomorphs were found, of which 17 were non-arboreal and 4 arboreal elements. Of the non-arboreal pollen, *Artemisia* had a relative abundance of 53.4% and Chenopodiaceae represented 18.3%. Asteraceae (1.9%), Fabaceae (1.7%), Ranunculaceae (1.7%), Caryophyllaceae (1.1%), Poaceae (0.7%), Lamiaceae (0.7%) and Brassicaceae (0.7%) were abundant elements. In addition, a small number of xerophilous *Ephedra* (0.5%) and aquatic *Typha* (0.1%) were also encountered. Arboreal pollen represented a total of 18.8%, including *Pinus* (16.9%), *Picea* (0.6%), *Betula* (1.2%), and *Juglans* (0.1%) (Fig. 2, Table 1).

#### Typical steppe

28 types of palynomorphs were identified, including 23 non-arboreal and 5 arboreal elements. The relative abundance of non-arboreal pollen was 88.8%, which were mainly composed of Chenopodiaceae (39%) and *Artemisia* (28.8%). Other abundant non-arboreal elements included Ranunculaceae (5.9%), Poaceae (2.9%), Caryophyllaceae (1.9%), Brassicaceae (1.6%), Asteraceae (1.5%) and Rosaceae (1.2%). In comparison with meadow steppe, the relative abundance of *Ephedra* increased (1.1%), while other xerophilous elements, i.e. *Tamarix* (0.8%) and Zygophyllaceae (0.1%) were present. Arboreal pollen constituted 11.2%, of which *Pinus* contributed 7.2% and *Betula* 3.4%. A few *Picea* (0.3%), *Juglans* (0.3%) and *Quercus* (0.1%) were also noted (Fig. 2, Table 1).

#### Desert steppe

This type of steppe yielded 25 types of palynomorphs, composed of 20 non-arboreal and 5 arboreal elements. Non-arboreal pollen were dominated by Chenopodiaceae (35.8%) and *Artemisia* (30.6%), while Ranunculaceae (4.1%), Asteraceae (2.4%), Caryophyllaceae (1%), Polygonaceae (0.8%), Poaceae (0.7%), Lamiaceae (0.7%), and Liliaceae (0.7%) were commonly observed. In comparison with typical steppe, the total relative abundance of xerophilous *Ephedra* (1.8%), *Tamarix* (2.2%) and Zygophyllaceae (5.8%) increased by almost 5 times. *Nitraria* was found only in desert steppe (4.7%). Arboreal pollen (7.8%) was mainly composed of *Pinus* (6.8%), *Abies* (0.6%) and *Picea* (0.3%) (Fig. 2, Table 1).

### 2. Pollen assemblages of warm temperate forest in Beijing, northern China

The surface pollen assemblage of warm temperate forest in Dongling Mountain, Beijing, northern China consisted of 33 types of palynomorphs, including 9 arboreal and 24 non-arboreal elements. The relative abundance of arboreal pollen was about 75.4%, of which *Pinus* contributed 64.5% to the total assemblage. *Juglans* (5.1%), *Betula* (4%) and *Corylus* (1%) were commonly encountered. Non-arboreal elements mainly comprised *Artemisia* (11.9%), Asteraceae (2.6%), Chenopodiaceae (2.4%) and Fabaceae (0.8%). The Poaceae had a relative abundance of 0.4%. A few xerophilous elements were found including *Ephedra* (0.2%), Zygophyllaceae (0.3%) and *Nitraria* (0.1%) (Table 2, S3 Table).

**Table 2:** The list of the palynomorphs and their relative abundances of temperate forest in Dongling Mountain, Beijing, northern China.

**Taxa**	**S RA (%)**	**A RA (%)**	**Taxa**	**S RA (%)**	**A RA (%)**
**Arboreal**			***Angiosperms***		
***Gymnosperms***			*Artemisia*	11.88	37.52
*Pinus*	64.54	19.95	Chenopodiaceae	2.36	7.58
*Abies*	0.44	0.66	Poaceae	0.44	2.47
*Platycladus*	0	0.03	Asteraceae	2.62	1.03
***Angiosperms***			Brassicaceae	0	0.86
*Juglans*	5.07	11.58	Fabaceae	0.79	0.26
*Betula*	4.02	11.56	Cyperaceae	0	0.08
*Quercus*	0	2.04	*Ricinus*	0	0.08
*Salix*	0	0.96	Rosaceae	0.35	0
*Ulmus*	0	0.95	*Sanguisorba*	0.17	0
*Corylus*	0.96	0.54	Araliaceae	0.09	0
Oleaceae	0.09	0.34	Caryophyllaceae	0.09	0
*Populus*	0	0.25	*Scabiosa*	0.09	0
*Tilia*	0.09	0.15	Gentianaceae	0.09	0
Sapindaceae	0	0.13	Boraginaceae	0	0.07
Anacardiaceae	0	0.09	Apiaceae	0.09	0.06
Ericaceae	0.09	0.07	Zygophyllaceae	0.26	0.05
*Ostryopsis*	0	0.06	Ranunculaceae	0.61	0.04
*Acer*	0	0.04	Rubiaceae	0	0.04
*Castanea*	0.09	0.04	Convolvulaceae	0	0.03
Rutaceae	0	0.03	*Humulus*	0.09	0.03
Elaeagnaceae	0	0.02	Lamiaceae	0	0.03
*Fraxinus*	0	<0.01	Pyrolaceae	0	0.03
**Non-arboreal**			*Tamarix*	0	0.03
***Pteridophytes***			*Nitraria*	0.09	0.03
Athyriaceae	0.35	0	Euphorbiaceae	0	0.02
Dennstaedtiaceae	0.09	0	Polygonaceae	0.17	0.02
*Selaginella*	0	0.05	Saxifragaceae	0	0.02
Polypodiaceae	0.17	<0.01	Campanulaceae	0	<0.01
*Pteris*	0.17	<0.01	Onagraceae	0	<0.01
Sinopteridaceae	3.23	<0.01	*Xanthium*	0	<0.01
***Gymnosperms***			**Algae**		
*Ephedra*	0.17	0.05	Zygnemataceae	0.17	<0.01

Note: S RA are the relative abundances of palynomorphs in surface pollen assemblages, where A RA are those in airborne pollen assemblages.

The airborne pollen assemblage of Dongling Mountain over the 4 years (2008–2011) contained 52 types of palynomorphs, including 21 arboreal and 31 non-arboreal elements. The relative abundance of arboreal pollen was about 50%, which was mainly composed of *Pinus* (20%), *Betula* (11.6%), and *Juglans* (11.6%). *Quercus* (2%), *Ulmus* (1%), *Salix* (1%) and *Corylus* (0.5%) were abundant elements. Of the non-arboreal elements, *Artemisia* (37%) and Chenopodiaceae (8%) were dominant. The Poaceae contributed a relative abundance of 2.5%, while Asteraceae (1%), Brassicaceae (0.9%) and Fabaceae (0.3%) were also found. The xerophilous elements *Ephedra* (0.05%), Zygophyllaceae (0.05%), *Tamarix* (0.03%) and *Nitraria* (0.03%) were rarely encountered (Table 2, S3 Table).

### 3. RDA ordination

In the RDA ordination of steppe samples (Fig. 3A), the first RDA axis explained 32% of the variation in the pollen data, and correlation coefficient between axis 1 and species-environmental variables was 0.608. The second axis accounted for only 2.9% of the variation, but it was well correlated with the environmental variables (*R* = 0.963). MAP, I_dm_ and longitude had high negative correlation coefficients (*R* = -0.8670, -0.9390, and -0.9286 respectively) with axis 2, while MAT showed a strong positive correlation with axis 2 (*R* = 0.8693). Therefore, it was reasonable to assume that axis 2 represented the gradient of aridity along longitude. A higher score on axis 2 might imply a more arid environment, and vice versa. Moreover, distribution of the pollen samples from different steppe types on axis 2 seemed to be consistent with the aridity gradient. Samples from meadow steppe had the lowest scores on axis 2, while those from desert steppe had the highest scores. Samples of typical steppe were located between those from meadow steppe and desert steppe, which reflected intermediate conditions.

Relative abundances of some pollen taxa were closely correlated with the aridity gradient (Fig. 3B). Most arboreal elements (e.g., *Betula*, *Juglans*, *Pinus*, and *Quercus*), non-arboreal mesophytes (e.g., Brassicaceae, Caryophyllaceae, Cyperaceae, Fabaceae, Poaceae, and Rosaceae) and hygrophytes (e.g., Cyperaceae) were located at the lower part of axis 2. This indicated that pollen relative abundances of these taxa increased with increasing humidity. In contrast, xerophilous elements like *Ephedra*, *Nitraria*, *Tamarix* and Zygophyllaceae occurred on the upper part of axis 2, and they showed strong negative correlations with MAP and I_dm_. This implied that the relative abundances of *Ephedra*, *Nitraria*, *Tamarix* and Zygophyllaceae increased with intensifying aridity.

Univariate linear regression analysis (Fig. 4A) showed a weak negative correlation (*R* ^*2*^ = 0.018, *P* = 0.646) between C/A ratio and aridity gradient (RDA axis 2). However, the total relative abundance of xerophilous elements (*Ephedra*, *Nitraria*, *Tamarix* and Zygophyllaceae) was significant, being positively related to the aridity gradient (Fig. 4B, *R* ^*2*^ = 0.889, *P* < 0.001).

In the RDA ordination including both steppe and forest samples (Fig. 3C), the first axis explained 25.2% of the variation in the pollen data, and axis 1 was also correlated with the environmental variables (*R* = 0.571). The second axis accounted for only 3.5% of the variation, but was highly correlated with environmental variables (*R* = 0.754). MAP, I_dm_ and longitude had moderate correlation coefficients (*R* = -0.5915, -0.6354, and -0.6320 respectively) with axis 2, which indicated that axis 2 to some extent could reflect the gradient of aridity. In this case, samples from warm temperate forest had the lowest scores on axis 2 and they were easily distinguished from steppe samples. Samples from meadow steppe and typical steppe had higher scores than forest, and samples from desert steppe had the highest scores of all.

## Discussion

### 1. Distinguishing 3 steppe types using surface pollen assemblages

We determined that surface pollen data could be used to differentiate the three types of steppe, and the four environmental variables explained the variation in pollen-based plant communities (Fig. 3A). Desert steppe samples were the most distinct. They were characterized by high relative abundances of xerophilous elements (such as *Ephedra*, *Tamarix*, *Nitraria*, and Zygophyllaceae). Meadow steppe samples showed low similarity with desert steppe samples and only had a small number of xerophilous elements. Samples from typical steppe were intermediate between desert steppe and meadow steppe samples. Noticeably, the boundary between samplesof meadow steppe and that of typical steppe were somewhat indistinct (Fig. 3A), especially when forest samples were included in the ordination analysis (Fig. 3C). Several reasons are possibly responsible for this phenomenon. Firstly, meadow steppe and typical steppe to some extent are similar in vegetation composition (S1 Table), thus their pollen assemblages might be difficult to distinguish from each other. Secondly, the algorithm of dimensionality reduction in ordination technique might distort some relationships among studied samples [47]. Thirdly, taxonomic resolution of pollen analysis (mostly at generic/family level) is not high enough to detect differences at the species level.

Variation in the pollen assemblages of different steppe types was correlated with four selected environmental variables (Fig. 3A). Desert steppe samples were related to low MAP, I_dm_ and longitude (west), but high MAT. On the contrary, Meadow steppe samples were correlated with high MAP, I_dm_ and longitude (east), but low MAT. Typical steppe samples were related to intermediate conditions between desert steppe and meadow steppe. The combined impact of these environmental variables appears to represent an aridity gradient (RDA axis 2). All of the four environmental variables contribute significantly to the aridity gradient, since they all were correlated with RDA axis 2. The distribution of pollen samples on this ordination gradient (Fig. 3A) reflects the actual aridity differences among habitats of three steppe types [17]. For example, desert steppe samples fell into the driest part of the gradient, while meadow steppe samples were in the wettest part. Therefore, pollen data have great potential to be used to indicate aridity changes in eastern Eurasian steppe.

The characteristics of pollen assemblage and some indicator taxa can be used to clearly delimit different steppe types. Meadow steppe, typical steppe and desert steppe form a gradient in Inner Mongolia as the aridity increases from east to west [17]. The xerophilous *Ephedra* was present in all three types of steppe, and its relative abundance increased in pollen assemblages as the aridity increased from meadow steppe to typical steppe and desert steppe. *Tamarix* and Zygophyllaceae only had a pollen record in typical steppe and desert steppe. In desert steppe, *Nitraria* was present as a unique arid indicator and the total relative abundance of *Ephedra*, *Tamarix* and Zygophyllaceae was also greater in this than other steppe types (Fig. 2, Table 1). Therefore, the three steppe types can be delimited based on the changes in the relative abundances of the pollen of these xerophilous elements. RDA ordination confirmed that there is a strong correlation between the relative abundances of *Ephedra*, *Tamarix*, *Nitraria*, Zygophyllaceae and the variation of MAP and I_dm_ (aridity index) with the highest relative abundance values of these taxa being found in the driest sites (Fig. 3B). Furthermore, total relative abundance of these xerophilous elements may be a potential aridity proxy in eastern Eurasian steppe, since it shows strong positive correlation with aridity gradient (Fig. 4B).

It is noteworthy that although the steppe of Inner Mongolia is grassland dominated by Poaceae [17], Poaceae had only a negligible relative abundance in the surface pollen assemblages (Fig. 2, Table 1). This phenomenon is consistent with previous investigations [13,15,16,18–22]. Surprisingly, the relative abundance of Poaceae was only 1.5% in steppe (Table 1) vs. 2.5% in forest (Table 2). This suggests that Poaceae pollen is not a useful indicator of steppe vegetation.

In addition, the steppe of Inner Mongolia was clearly distinguished from the warm temperate forest of Dongling Mountain based on the ordination analysis of the pollen data. In the RDA biplot (Fig. 3C), airborne and surface pollen samples from the forest were both located in the bottom right quadrant and clearly distinct from the steppe samples. Furthermore, the forest samples had lower scores on RDA axis 2 corresponding to more humid conditions than steppe. Several pollen indicators can be used to distinguish forest vs. steppe. For example, the arboreal elements in temperate forest surface/airborne pollen assemblages were greater than among pollen assemblages from steppe, although *Pinus* and *Betula* were the foremost arboreal elements in both steppe and forest. On the contrary, non-arboreal elements dominated pollen assemblages from steppe. Chenopodiaceae and *Artemisia* were over-represented non-arboreal elements, and they had high relative abundances in both steppe and forest. Besides, the xerophilous *Ephedra*, *Nitraria*, *Tamarix* and Zygophyllaceae had much higher relative abundances in steppe than in forest.

### 2. Correlation between Chenopodiaceae/*Artemisia* ratio (C/A) and aridity

To date, several studies have indicated the varying importance of the C/A ratio as an indicator of aridity [13,15,16,18–20,28]. Here we observed that the C/A ratio was not correlated (*R* ^*2*^ = 0.018) with aridity gradient (Fig. 4A), although Chenopodiaceae and *Artemisia* were dominant elements in surface pollen assemblages from all three types of steppe (Table 1). The C/A ratio of meadow steppe can occasionally be greater than that of desert steppe, and the mean C/A ratio of typical steppe was the greatest (Fig. 2, Table 1). This confirms that the C/A ratio could not be used as a proxy for aridity changes in the eastern Eurasian steppe.

The inconsistency of C/A ratio as a predictor of aridity likely relates to geographical variation in communities and prevalence of specific Chenopodiaceae and *Artemisia* taxa. For example, the Chenopodiaceae is a diverse family with 103 genera and 1300 species, and *Artemisia* is a diverse genus with 350 species [48]. However, the individual species per family or genus can occupy varying niches with different aridities. For instance while most *Artemisia* grow in arid conditions, *Artemisia selengensis* can live along lake shores, in wetland, or even in shallow water as an emergent plant [49]. Such inconsistencies likely contribute to the varying strength of correlations between C/A ratio and aridity.

Our results also indicate that both Chenopodiaceae and *Artemisia* were dominant non-arboreal elements in the pollen assemblages of both steppes (Table 1) and northern temperate forest (Table 2). They represented a total relative abundance of 69% in the surface pollen assemblage of steppe, and 45% in the airborne pollen assemblage of northern temperate forest. It is obvious that different species of Chenopodiaceae and *Artemisia* were involved (in the steppe and forest). Therefore, using taxa occupying such diverse niches to indicate aridity may not be appropriate. It is clearly better to choose those taxa with only a limited number of species and restricted niches to indicate aridity changes in the eastern Eurasian steppe, such as *Ephedra*, *Nitraria*, *Tamarix* and Zygophyllaceae.

## Supporting Information

S1 Fig Main palynomorphs of Inner Mongolian steppe in eastern Eurasia. 1. *Pinus*; 2. *Betula*; 3. *Juglans*; 4. *Artemisia*; 5. Chenopodiaceae; 6. Fabaceae; 7. Poaceae. 8. Caryophyllaceae; 9. Apiaceae; 10. Asteraceae; 11. Lamiaceae; 12. Rosaceae; 13. Brassicaceae; 14. Rubiaceae; 15. *Typha*; 16. *Nitraria*; 17. Zygophyllaceae; 18. *Tamarix*; 19. *Ephedra*. Scale bar = 10 μm.
(TIF)

S1 Table Distribution of plant taxa in three steppe types of Inner Mongolia, China, based on the pollen resolution. (DOC)

S2 Table The list of plant taxa of temperate forest in Dongling Mountain, Beijing, China, based on the pollen resolution. (DOC)

S3 Table Pollen assemblages, locations and climatic information for studied sampling sites in Inner Mongolia and Dongling Mountain. (XLS)
