# Supplementary figures and images for: Utility of Surface Pollen Assemblages to Delimit Eastern Eurasian Steppe Types
Source: PLoS One. 2015 Mar 12;10(3):e0119412. doi: 10.1371/journal.pone.0119412 (PMC4357446; doi:10.1371/journal.pone.0119412)

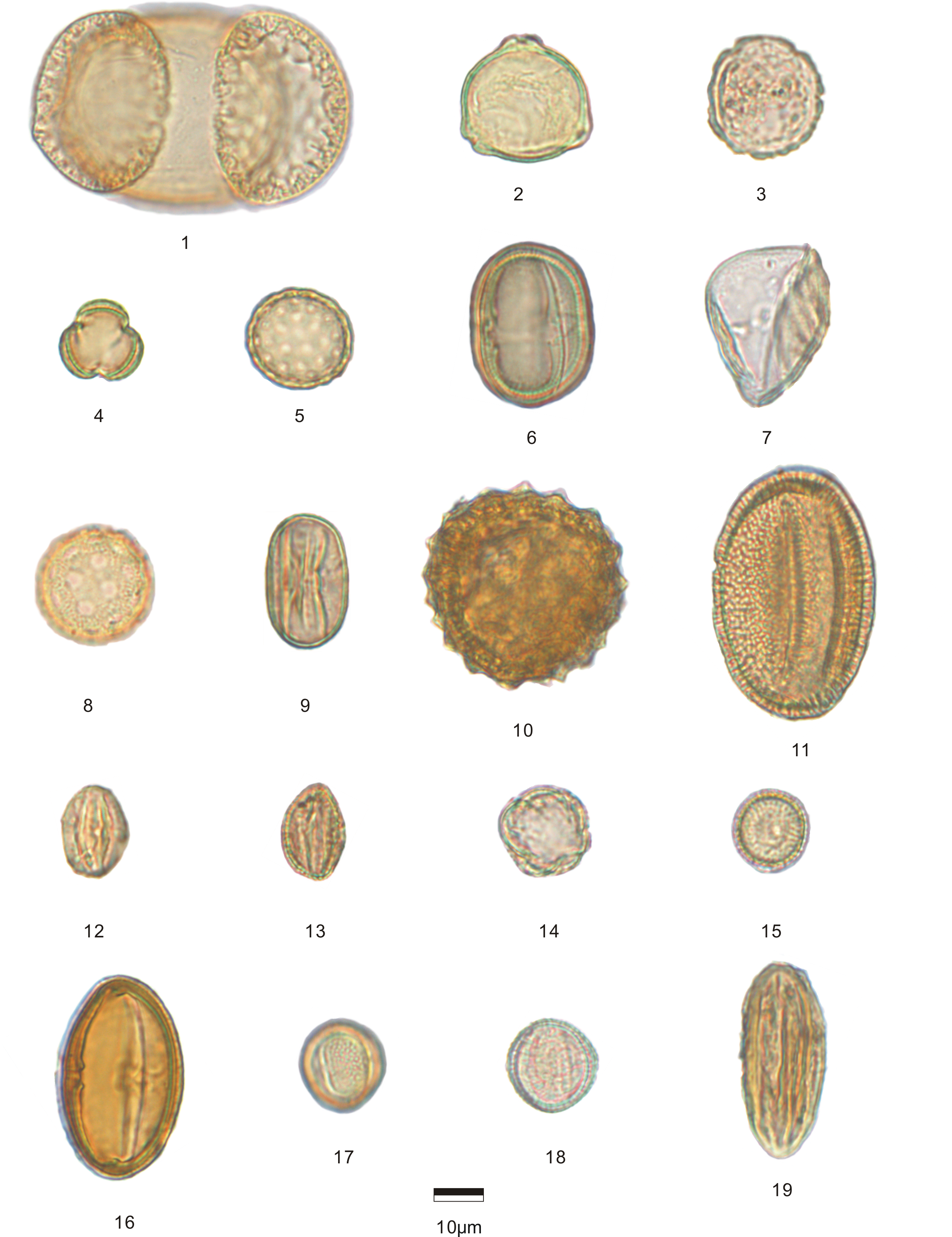

Supplement: S1 Fig — 1. Pinus; 2. Betula; 3. Juglans; 4. Artemisia; 5. Chenopodiaceae; 6. Fabaceae; 7. Poaceae. 8. Caryophyllaceae; 9. Apiaceae; 10. Asteraceae; 11. Lamiaceae; 12. Rosaceae; 13. Brassicaceae; 14. Rubiaceae; 15. Typha; 16. Nitraria; 17. Zygophyllaceae; 18. Tamarix; 19. Ephedra. Scale bar = 10 μm. (TIF) [file pone.0119412.s001.tif]
