# Supplementary material for: Utility of Surface Pollen Assemblages to Delimit Eastern Eurasian Steppe Types
Source: PLoS One. 2015 Mar 12;10(3):e0119412. doi: 10.1371/journal.pone.0119412 (PMC4357446; doi:10.1371/journal.pone.0119412)
Supplement: S1 Table — (DOC) [file pone.0119412.s002.doc]

**Table S1. Distribution of plant taxa in three steppe types of Inner Mongolia, China, based on the pollen resolution.**

|  | **MS** | **TS** | **DS** |  | **MS** | **TS** | **DS** |
| --- | --- | --- | --- | --- | --- | --- | --- |
| **Poaceae** § | ● | ● | ● | **Primulaceae** ? * | ● | ● | ● |
| **Asteraceae** § † | ● | ● | ● | **Gentianaceae** ? * | ● | ● | ● |
| *Artemisia* **(Asteraceae)** § | ● | ● | ● | **Crassulaceae** ? * | ● | ● | ● |
| **Fabaceae** §* | ● | ● | ● | **Euphorbiaceae** ? † | ● | ● | ● |
| **Rosaceae** § * | ● | ● | ● | **Polygalaceae** ? † | ● | ● | ● |
| **Chenopodiaceae** § | ● | ● | ● | Asclepiadoideae **(**[**Apocynaceae**](http://en.wikipedia.org/wiki/Apocynaceae)**)** ? * | ● | ● | ● |
| **Liliaceae** §* | ● | ● | ● | **Campanulaceae** ? * | ● | ● | ● |
| **Cyperaceae** § | ● | ● | ● | **Thymelaeaceae** ? * | ● | ● | ● |
| **Apiaceae** * | ● | ● | ● | **Geraniaceae** ? * | ● | ● |  |
| **Brassicaceae** * | ● | ● | ● | **Loganiaceae**? * | ● | ● |  |
| **Lamiaceae** * | ● | ● | ● | **Berberidaceae** ? * | ● | ● |  |
| **Caryophyllaceae** * | ● | ● | ● | **Clusiaceae** ? * | ● |  |  |
| **Scrophulariaceae** * | ● | ● | ● | **Rhamnaceae** ? * | ● |  |  |
| **Ranunculaceae** * | ● | ● |  | **Cupressaceae** ? | ● |  |  |
| **Rubiaceae** * | ● | ● |  | **Iridaceae** ? * | ● |  | ● |
| *Viola* **(Violaceae)** * | ● | ● |  | **Linaceae** ? * | ● |  | ● |
| **Caprifoliaceae** * | ● | ● |  | **Plumbaginaceae** ? * | ● |  | ● |
| **Polygonaceae** † | ● | ● |  | **Rutaceae** ? * |  | ● | ● |
| **Boraginaceae** * |  | ● | ● | **Convolvulaceae** ? * |  | ● | ● |
| *Betula* **(Betulaceae**) |  | ● |  | **Verbenaceae** ? * |  | ● | ● |
| *Ephedra* **(Ephedraceae)** ‡ | ● | ● | ● | **Oleaceae** ? * |  | ● |  |
| **Tamaricaceae** ‡* |  |  | ● | **Saxifragaceae** ? * |  | ● |  |
| **Zygophyllaceae** ‡* |  |  | ● | **Orobanchaceae** ? * |  | ● |  |
| *Nitraria* **(Nitrariaceae)** ‡* |  |  | ● | *Ostryopsis***(Betulaceae)** ? |  | ● |  |
|  |  |  |  | **Plantaginaceae** ? |  |  | ● |

Note: § indicates the dominant taxa; ‡ represents the xerophilous elements; ? indicates the pollen signals of the taxa are not detected in the surface pollen assemblages; ● indicates the taxa are present in the steppe type; MS, meadow steppe; TS, typical steppe; DS, desert steppe; * indicates entomophilous taxon; † shows plant taxon with entomophilous and anemophilous habits. Data are cited from our field investigation, Wu [42] and ISTIMN [17].
