# Supplementary material for: Utility of Surface Pollen Assemblages to Delimit Eastern Eurasian Steppe Types
Source: PLoS One. 2015 Mar 12;10(3):e0119412. doi: 10.1371/journal.pone.0119412 (PMC4357446; doi:10.1371/journal.pone.0119412)
Supplement: S2 Table — (DOC) [file pone.0119412.s003.doc]

**Table S2. The list of plant taxa of temperate forest in Dongling Mountain, Beijing, China, based on the pollen resolution.**

| **Pteridophytes** | *Castanea* | Meliaceae | Boraginaceae |
| --- | --- | --- | --- |
| Selaginellaceae | *Quercus* § | Polygalaceae | Verbenaceae |
| Equisetaceae | *Celtis* † | Euphorbiaceae | Lamiaceae |
| Botrychiaceae | *Ulmus* † | Anacardiaceae | Solanaceae |
| Dennstaedtiaceae | *Hemiptelea* | Celastraceae ‡ | Scrophulariaceae |
| Pteridiacaee | Cannabaceae | Sapindaceae | Bignoniaceae |
| Sinopteridaceae | *Morus* (Moraceae) | *Acer* (Sapindaceae) † | Pedaliaceae |
| Hemionitidaceae | Urticaceae | Balsaminaceae | Orobanchaceae |
| Athyriaceae | Santalaceae | Rhamnaceae | Gesneriaceae |
| Aspleniaceae | Aristolochiaceae | Vitaceae | Plantaginaceae |
| Onocleaceae | Loranthaceae | *Tilia* (Tiliaceae) † | Rubiaceae |
| Woodsiaceae | Polygonaceae | Tiliaceae | Phrymaceae |
| Dryopteridaceae | Amaranthaceae | Malvaceae | Caprifoliaceae ‡ |
| Polypodiaceae | Portulacaceae | Actinidiaceae | Hydrangeaceae ‡ |
| **Gymnosperms** | Caryophyllaceae | Clusiaceae | Adoxaceae |
| *Ginkgo* | Ranunculaceae ‡ | *Viola* | Cucurbitaceae |
| *Abies* | Berberidaceae | Begoniaceae | Campanulaceae |
| *Cedrus* | Menispermaceae | Thymelaeaceae | Asteraceae ‡ |
| *Larix* † | Magnoliaceae | Elaeagnaceae | *Artemisia* (Asteraceae) |
| *Picea* | Papaveraceae | Onagraceae | Juncaginaceae |
| *Pinus* † | Brassicaceae | Araliaceae | Poaceae ‡ |
| Cupressaceae | Crassulaceae | Apiaceae | Cyperaceae ‡ |
| *Ephedra* | Saxifragaceae ‡ | Cornaceae | Araceae |
| **Angiosperms** | Rosaceae ‡ | Ericaceae ‡ | Commelinaceae |
| Chloranthaceae | *Sanguisorba* (Rosaceae) | Primulaceae | Juncaceae |
| *Populus* § | Fabaceae ‡ | Ebenaceae | Liliaceae ‡ |
| *Salix* † | Oxalidaceae | Oleaceae ‡ | Dioscoreaceae |
| *Juglans* § | Geraniaceae | *Fraxinus* (Oleaceae) † | Iridaceae |
| *Betula* § | Linaceae | Gentianaceae | Orchidaceae |
| *Carpinus* | Zygophyllaceae | Asclepiadoideae (Apocynaceae) |  |
| *Corylus* ‡ | Rutaceae | Convolvulaceae |  |
| *Ostryopsis* | Simaroubaceae | Polemoniaceae |  |

Note: § indicates the dominant taxa; † indicates the abundant taxa of tree layer; ‡ indicates the abundant taxa of shrub and herb layer. Data are cited from our field investigation and “The list of plant taxa at the Xiaolongmen Area, Beijing, China”
